# Insight into the Karyotype Evolution of *Brachypodium* Species Using Comparative Chromosome Barcoding

**DOI:** 10.1371/journal.pone.0093503

**Published:** 2014-03-27

**Authors:** Dominika Idziak, Iwona Hazuka, Beata Poliwczak, Anna Wiszynska, Elzbieta Wolny, Robert Hasterok

**Affiliations:** Department of Plant Anatomy and Cytology, Faculty of Biology and Environmental Protection, University of Silesia in Katowice, Katowice, Poland; Leibniz-Institute of Plant Genetics and Crop Plant Research (IPK), Germany

## Abstract

Paleogenomic studies based on bioinformatic analyses of DNA sequences have enabled unprecedented insight into the evolution of grass genomes. They have revealed that nested chromosome fusions played an important role in the divergence of modern grasses. Nowadays, studies on karyotype evolution based on the sequence analysis can also be effectively complemented by the fine-scale cytomolecular approach. In this work, we studied the karyotype evolution of small genome grasses using BAC-FISH based comparative chromosome barcoding in four *Brachypodium* species: diploid *B. distachyon* (2n = 10) and *B. sylvaticum* (2n = 18), diploid (2n = 18) and allopolyploid (2n = 28) *B. pinnatum* as well as *B. phoenicoides* (2n = 28). Using BAC clones derived from the *B. distachyon* genomic libraries for the chromosomes Bd2 and Bd3, we identified the descending dysploidy events that were common for diploids with x = 9 and *B. distachyon* as well as two nested chromosome fusions that were specific only for *B. distachyon*. We suggest that dysploidy events that are shared by different lineages of the genus had already appeared in their common ancestor. We also show that additional structural rearrangements, such as translocations and duplications, contributed to increasing genome diversification in the species analysed. No chromosomes structured exactly like Bd2 and Bd3 were found in *B. pinnatum* (2n = 28) and *B. phoenicoides*. The structure of Bd2 and Bd3 homeologues belonging to the two genomes in the allopolyploids resembled the structure of their counterparts in the 2n = 18 diploids. These findings reinforce the hypothesis which excludes *B. distachyon* as a potential parent for Eurasian perennial *Brachypodium* allopolyploids. Our cytomolecular data elucidate some mechanisms of the descending dysploidy in monocots and enable reconstructions of the evolutionary events which shaped the extant karyotypes in both the genus *Brachypodium* and in grasses as a whole.

## Introduction

The enormous diversity of angiosperm plants is, to a large extent, a reflection of the great variation in their genomes. This is manifested through striking differences in genome size and in the number, size and morphology of the chromosomes that constitute their karyotypes [1]. Recent data indicate that 1C-DNA values among angiosperms range from about 0.065 pg to more than 152 pg [2]. A similar variation is also observed in the chromosome number.

The most important mechanisms which determine a numerical variation of chromosomes in plants are polyploidisation and dysploidy [3]. Recent data indicate that polyploidisation is even more frequent and ubiquitous among angiosperms than was previously supposed [4]. The occurrence of several ancient genome doubling events that are common to all angiosperms and lineage-specific whole genome duplications were recently documented. This infers that polyploidy is a major evolutionary force driving the success of flowering plants [4]–[7]. Dysploidy may alter not only the number of chromosomes but also their size and shape [1], [8]. Whole genome duplication and dysploidy are often accompanied by additional minor structural rearrangements, which do not change the chromosome number but do contribute to karyotype variation. Taken together, these phenomena are the forces that shape the karyotype structure and drive their evolution, and presumably also play a significant role in speciation [3].

Studies of the organisation of karyotypes can provide a plethora of data with a vast range of applications, such as whole genome sequencing projects [9]–[11], breeding programmes [12] and phylogenetic studies [13]–[15]. It also forms the basis for paleogenomics, and thus enables deduction about the structure of the ancestral karyotype of investigated taxa and the reconstruction of the sequence of events that shaped the extant karyotypes [3], [7].

There are several approaches to the analysis of karyotype evolution which, along with additional support from molecular phylogenetics, result in comprehensive studies of genome diversification and speciation. One is based on comparative genetic and physical mapping. Marker-based collinearity studies permit the alignment of chromosomal segments in different cereal genomes and visualise them in the form of syntenic chromosomal regions arranged in concentric circles [16], [17]. However, genetic mapping has its limitations, such as the necessity of large mapping populations and frequent discrepancies between the genetic and physical maps [18].

Rapidly growing resources, such as whole genome sequence and expressed sequence tag (EST) databases can be employed to assess the evolutionary relationship between the extant angiosperm karyotypes and deduced ancestral genomes. The number of protochromosomes in ancestral karyotypes was inferred and possible evolutionary scenarios were proposed for both eudicot and monocot lineages [5], [7], [19], [20]. Bioinformatic analyses of grass genome sequences and ESTs combined with high resolution genetic mapping and earlier macrocolinearity studies led to the significant updating of the original ‘crop circle’ [17] by including chromosomal data of newly studied grass genomes and putative monocot ancestors [16], [21].

Another approach to uncover the origins of chromosome structure and karyotype evolution is cytomolecular mapping, which utilises libraries of large inserts of genomic DNA that are cloned into high capacity vectors, usually bacterial artificial chromosomes (BACs) together with fluorescence *in situ* hybridisation (FISH). This has the advantage of the direct visualisation and analysis of the chromosomal rearrangements that are responsible for karyotype differentiation in related genomes. Such studies are particularly advanced for the Brassicaceae family, where comparative chromosome painting (CCP) based on the cross-species mapping of *Arabidopsis thaliana* BAC contigs by FISH helped to describe the mechanisms of descending dysploidy in this group of species [22]. BAC-FISH-based chromosome barcoding in seven *Solanum* species of the Solanaceae revealed peri- and paracentric inversions that affects chromosome 6 in specific lineages of the genus and thus enabled the construction of the ancestral chromosome [23], [24].

Cytomolecular karyotype analyses of eudicots efficiently complement data obtained from genetic mapping and DNA sequence analysis, although similar studies in monocots are rather scarce in comparison. Presumably, this is due to the fact that cereal species are much less tractable for cytomolecular mapping due to their large and highly repetitive DNA nuclear genomes.

Establishing a weedy grass, *Brachypodium distachyon*, as a model for monocots changed this situation significantly. The feasibility of mapping *B. distachyon* BACs in the chromosomes of its close relatives was demonstrated previously [25]–[27]. Importantly, the genus *Brachypodium* represents a particularly suitable model system for the analysis of grass karyotype evolution. It comprises 14–19 species with different basic chromosome numbers of 5, 7, 8, 9 and 10, and different ploidy levels. *Brachypodium* karyotypes also display some differences in chromosome size and morphology [28]–[31]. These features make the *Brachypodium* species ideal as a model system that could be useful in elucidating the mechanisms of the chromosome rearrangements that are responsible for the divergence of grass genomes.

In this study we extend our previous, general comparative cytomolecular analysis of the genus *Brachypodium* to a much more detailed comparative chromosome barcoding by BAC-FISH applied to the chromosomes of four *Brachypodium* species: diploid *B. distachyon* (2n = 10), *B. sylvaticum* (2n = 18), diploid and allopolyploid *B. pinnatum* (2n = 18 and 2n = 28) and allopolyploid *B. phoenicoides* (2n = 28). The genome of *B. distachyon* comprises five chromosomes, most of which are morphometrically different. We focused on the analysis of the BAC clones derived from the chromosomes Bd2 and Bd3 because despite the almost identical size and shape of these chromosomes, they arose through a different number of nested fusions from the hypothetical intermediate grass ancestor that is characterised by a basic chromosome number of 12.

## Results

The karyotypes of three *Brachypodium* species, both diploids and allopolyploids (Table 1), were compared with reference to the model karyotype of *B. distachyon* using a heterologous BAC-FISH mapping (chromosome barcoding) approach. For these analyses, 17 BAC clones that contained mostly unique sequences were used. All of the clones were derived from the FingerPrinted Contigs (FPC) that had previously been assigned to the chromosomes Bd2 (11 clones) and Bd3 (6 clones) of *B. distachyon* [26], [32]. According to the FPC-derived physical map, these clones span the entire length of their respective chromosomes (Table 2).

**Table 1:** Origins, chromosome numbers and accession details of the *Brachypodium* species studied.

Species	Accession number	2n	Origin
*B. distachyon*	Bd21 (PI 254867)	10	Iraq
*B. sylvaticum*	PI 297868	18	Australia
*B. pinnatum*	PI 230113	18	Iran
	PI 430277	28	Ireland
*B. phoenicoides*	PI 253503	28	Spain

**Table 2:** Specification of BAC clones used for comparative chromosome barcoding.

BAC number	BAC clone identifier*	Position in genome (bp)
Bd2/1	BD_ABa0026H23	Bd2: 501743 : 631176
Bd2/2	BD_CBa0048M15	Bd2: 3999943 : 4170302
Bd2/3	BD_ABa0044B16	Bd2: 8154609 : 8305485
Bd2/4	BD_ABa0005E09	Bd2: 10380990 : 10507985
Bd2/5	BD_CBa0023P23	Bd2: 13336480 : 13486307
Bd2/6	BD_ABa0026K14	Bd2: 19861012 : 20005795
Bd2/7	BD_ABa0014K11	Bd2: 34309867 : 34503922
Bd2/8	BD_CBa0016E24	Bd2: 39997753 : 40003453
Bd2/9	BD_CBa0031I09	Bd2: 46500135 : 46639653
Bd2/10	BD_ABa0031O24	Bd2: 52001822 : 52162247
Bd2/11	BD_ABa0038G14	Bd2: 57002804 : 57148130
Bd3/1	BD_ABa0024P19	Bd3: 1507465 : 1643914
Bd3/2	BD_ABa0029A17	Bd3: 7006740 : 7159041
Bd3/3	BD_CBa0014A01	Bd3: 20363699 : 20508591
Bd3/4	BD_CBa0007K04	Bd3: 34625943 : 34771746
Bd3/5	BD_ABa0019B17	Bd3: 50354409 : 50508627
Bd3/6	BD_CBa0037C16	Bd3: 57302467 : 57322111

* More details on the clones used can be found in the NCBI database under the following URLs: http://www.ncbi.nlm.nih.gov/clone/library/genomic/424/ (BD_ABa library) and http://www.ncbi.nlm.nih.gov/clone/library/genomic/426/ (BD_CBa library).

**Figure 1:**
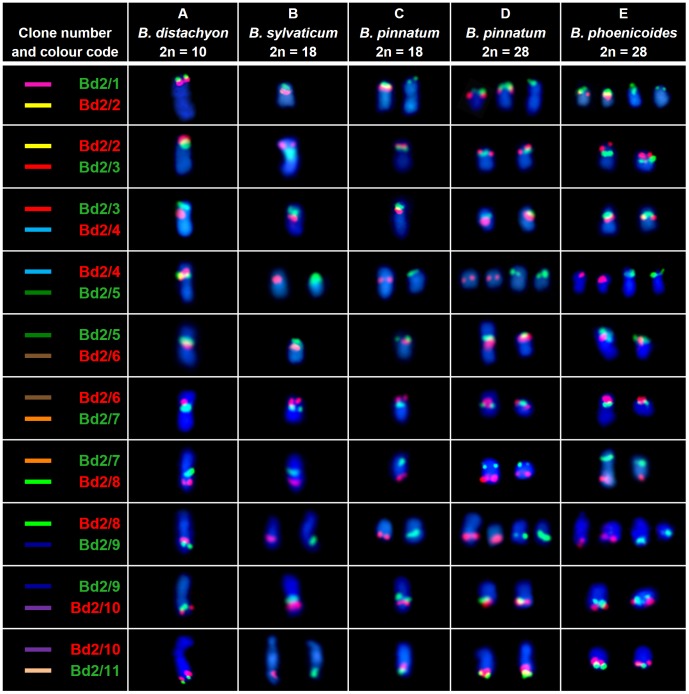
Comparative BAC-FISH mapping of clones from the chromosome Bd2 to various species of *Brachypodium*. Only one chromosome of a homologous pair is shown in each cell. The colour of the text label in the first column indicates the fluorochrome used (red – tetramethylrhodamine, green – FITC). The coloured bars assigned to specific clones correspond to their positions marked on cytogenetic maps in Figures 4 and 6. Chromosomes were counterstained with DAPI.

**Figure 2:**
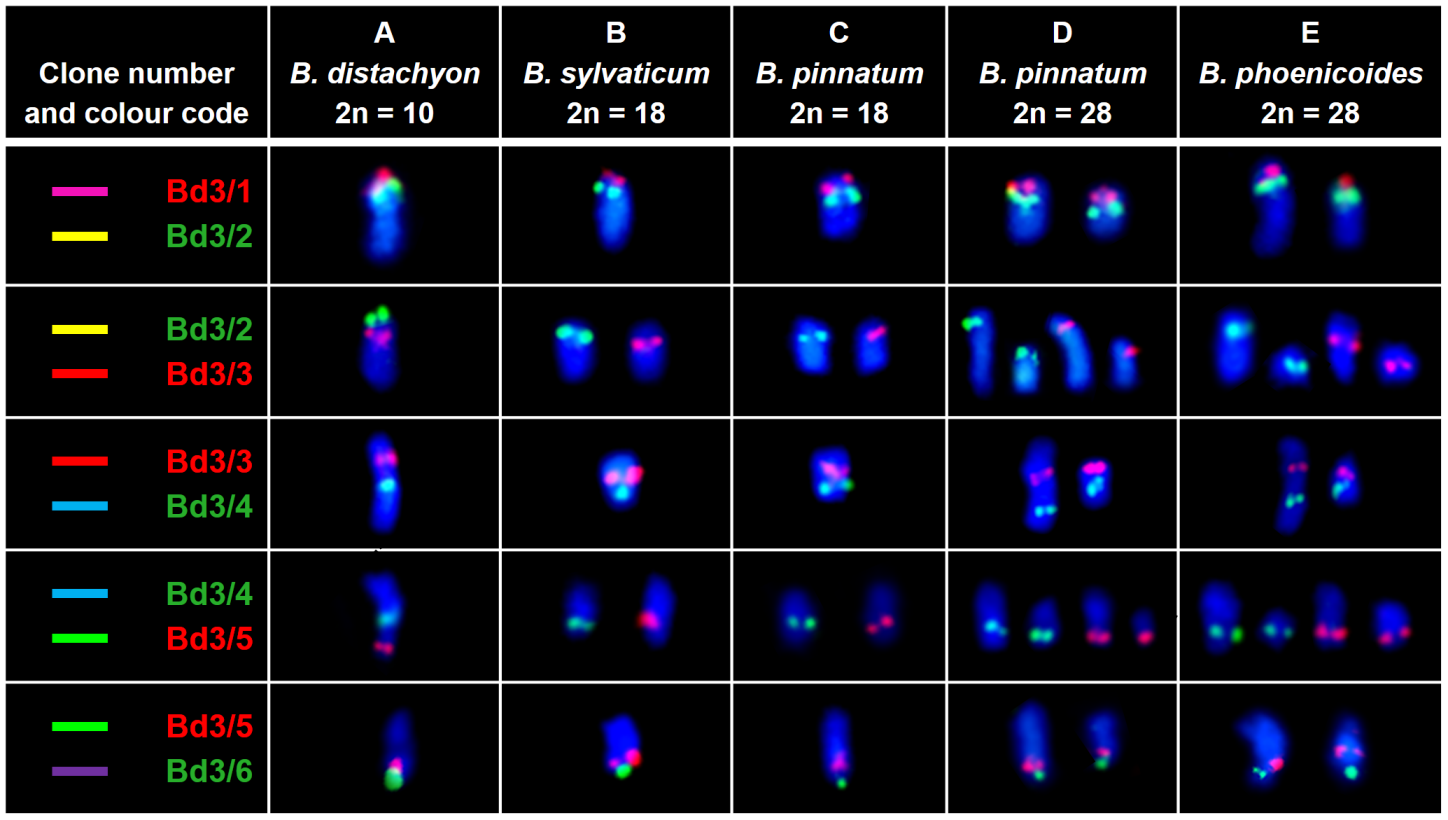
Comparative BAC-FISH mapping of clones from the chromosome Bd3 to various species of *Brachypodium*. Only one chromosome of a homeologous pair is shown in each cell. The colour of the text label in the first column indicates the fluorochrome used (red – tetramethylrhodamine, green – FITC). The coloured bars assigned to specific clones correspond to their positions marked on cytogenetic maps in Figures 5 and 7. Chromosomes were counterstained with DAPI.

For the principal FISH experiments, each of the clones was paired with a differently labelled clone that occupied an adjacent position on the physical map of a given chromosome. In supplementary BAC-FISH analyses, other combinations of clones were also used to fully resolve the relationship between the mapped chromosome regions. The hybridisation sites of the selected clones corresponded to their predicted positions on the physical map of *B. distachyon* (Figures 1A and 2A). Cross-species mapping revealed homeologues of chromosomes Bd2 and Bd3 of *B. distachyon* in all of the species studied.

### BACs derived from chromosome Bd2

**Figure 3:**
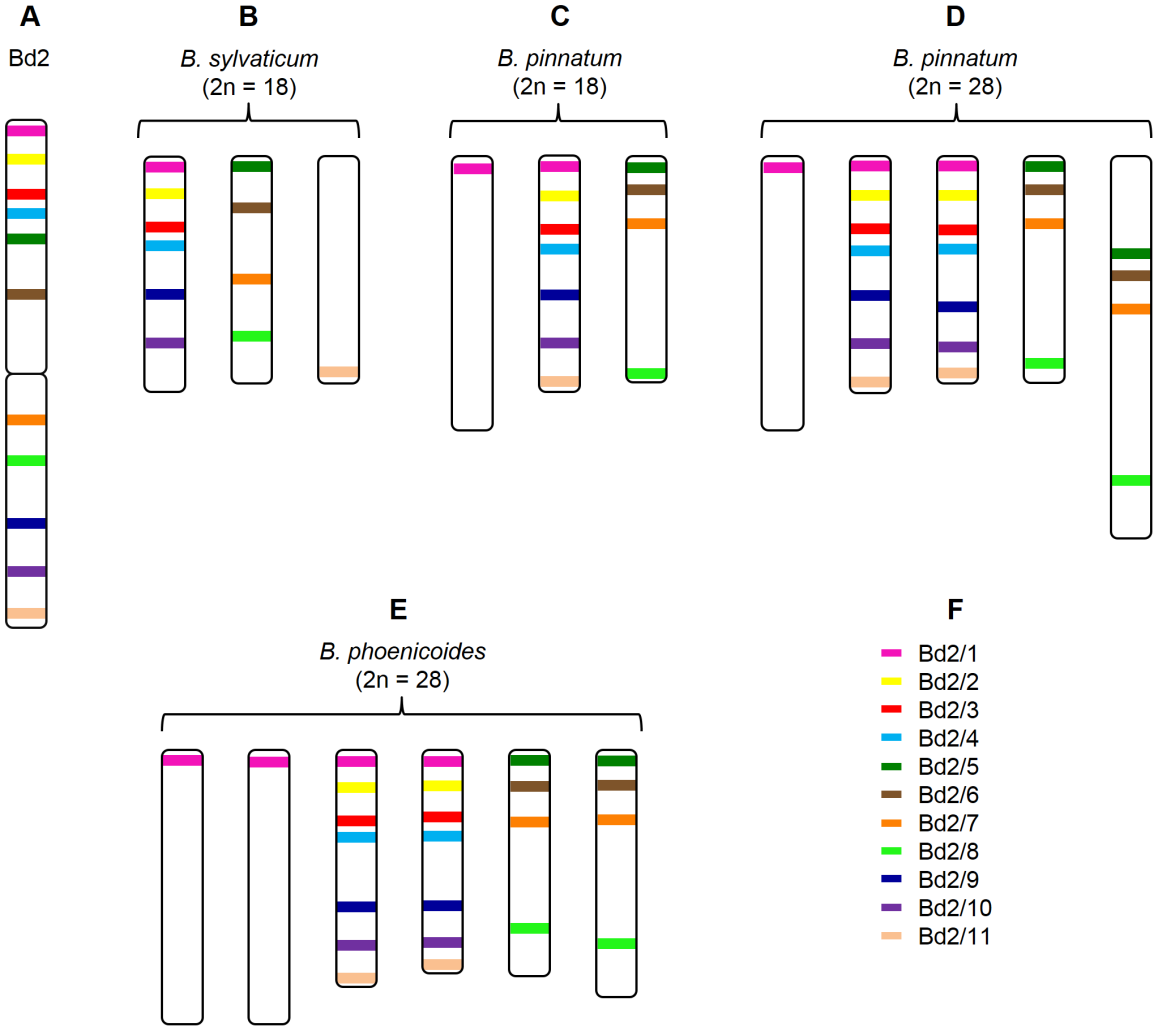
Cytogenetic maps of chromosomes bearing regions homeologous to chromosome Bd2 in various *Brachypodium* species. Coloured bars mark the chromosomal positions of specific BAC clones assigned to the chromosome Bd2 in *B. distachyon* (**A**), *B. sylvaticum* (**B**), *B. pinnatum* 2n = 18 (**C**), *B. pinnatum* 2n = 28 (**D**), *B. phoenicoides* (**E**). Colour codes for the clones used in the study (**F**). The position of the clones on the diagram (**A**) reflects their localisation on the physical map [32].

The number of chromosomes highlighted by the combination of clones Bd2/1-4 varied among the species investigated. All of the clones hybridised to a single chromosome pair in *B. sylvaticum* (Figure 1B), while two chromosome pairs were revealed in the diploid (2n = 18) *B. pinnatum*, one carrying four hybridisation signals and one with the signal only for the clone Bd2/1 (Figure 1C). Two pairs of chromosomes with hybridisation sites for all of the probes were found in the allopolyploid (2n = 28) cytotype of *B. pinnatum* and in *B. phoenicoides* (Figure 1 and 3). Additionally, *B. pinnatum* (2n = 28) had one chromosome pair carrying the signal for Bd2/1 while *B. phoenicoides* had two such pairs (Figure 1D and 1E, respectively). In all the species analysed, the chromosomes bearing signals were significantly smaller than their counterparts in the reference karyotype of *B. distachyon*. The order and orientation of these four clones were the same as in *B. distachyon*.

The heterologous mapping of probes Bd2/4 and Bd2/5 together showed that these two clones always land on separate chromosome pairs (Figure 1B–1E). As expected, the number of clone Bd2/5 signals in the allopolyploids was twice that of *B. sylvaticum* and *B. pinnatum* 2n = 18. In contrast to *B. distachyon*, where Bd2/5 occupies a proximal position in the top arm of chromosome Bd2, it maps terminally in other diploid species (Figure 1A–1C). In *B. phoenicoides* and allopolyploid *B. pinnatum*, the smaller of the two chromosome pairs that were discriminated had a terminal site for Bd2/5, while the larger pair had proximal probe signals (Figure 1D–1E).

Although clones Bd2/5, Bd2/6, Bd2/7 and Bd2/8 hybridise to the same chromosomes in all of the species analysed, their distribution pattern is different both in terms of the number and chromosomal position of the signals (Figures 1 and 3). The order of the clones resembled that of *B. distachyon* in both the diploids and allopolyploids. However, in *B. sylvaticum* clones Bd2/7 and Bd2/8 map to the same chromosome arm, which is different to the one carrying sites for Bd2/5 and Bd2/6, while in *B. pinnatum* (2n = 18) Bd2/7 and Bd2/8 land in opposite arms (Figure 1B–1C). In the latter species, Bd2/7 maps together with Bd2/5 and Bd2/6. These results suggest that the genomes of diploids might be differentiated by a pericentric inversion that contains the sequence of Bd2/7. In the allopolyploid *B. pinnatum*, the smaller pair of chromosomes that is marked by clones Bd2/5-8 has the same pattern as the diploid cytotype of this species. The probes span the entire chromosome from one end to the other. In contrast, the same set of clones spread from the proximal region of one arm to the terminal region of the opposite arm of the larger pair (Figure 1D). In *B. phoenicoides*, both chromosome pairs have the same distribution of clones as in diploid *B. pinnatum* (Figure 1E).

Heterologous BAC-FISH showed that clones Bd2/9 and Bd2/10 map to the same chromosomes, which are different from those discriminated by the probes Bd2/5-8 (Figure 1). One or two pairs of chromosomes carrying the signals were observed in the diploid and allopolyploid species, respectively. In the diploids and in one of the two chromosome pairs of the allopolyploids, probe Bd2/9 hybridises to a proximal region, while the Bd2/10 site is located in an intercalary position in the same arm. In the second pair of chromosomes in *B. phoenicoides* and *B. pinnatum* (2n = 28) karyotypes, the position of both probes is shifted towards the end of the chromosome. In most cases Bd2/11 maps terminally to the chromosomes with Bd2/10. A remarkable exception is seen in *B. sylvaticum*, where the signal of Bd2/11 is in the terminal position on a different chromosome pair (Figure 1B).

**Figure 4:**
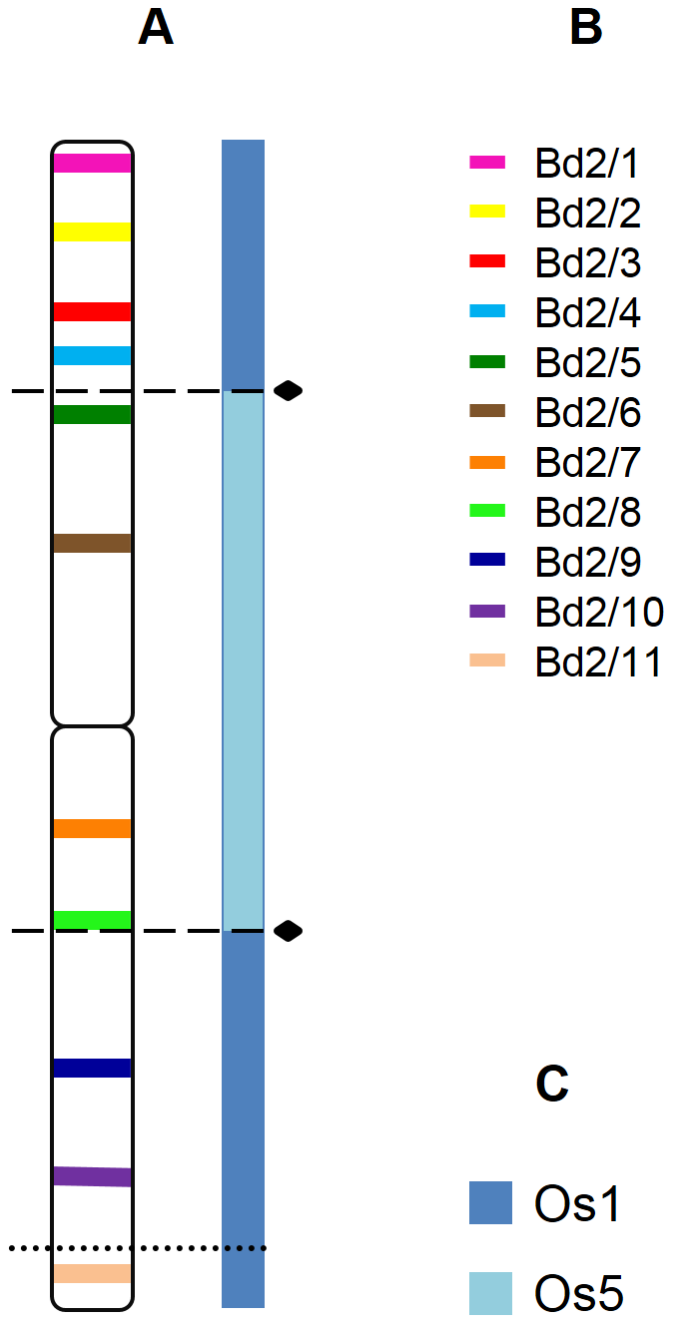
Positions of clones used in reference to the regions of the chromosome Bd2 homeologous with rice chromosomes. Coloured bars mark the chromosomal positions of specific BAC clones assigned to the chromosome Bd2. A cytogenetic map of *B. distachyon* chromosome Bd2 linked with the Bd2 assembly schematics showing the regions that correspond to different rice chromosomes (**A**) (adapted from [9]), Colour codes for the clones used in the study (**B**), Colour codes for rice chromosomes homologous to the chromosome Bd2 (**C**). Black diamonds identify the positions of the fusion points in the Bd2. Dashed lines mark the chromosomal breakpoints found in *B. sylvaticum*, *B. pinnatum* 2n = 18 and 2n = 28, and in *B. phoenicoides*. A dotted line marks the breakpoint specific for *B. sylvaticum*. The position of the clones on the diagram (**A**) reflects their localisation on the physical map [32].

**Figure 5:**
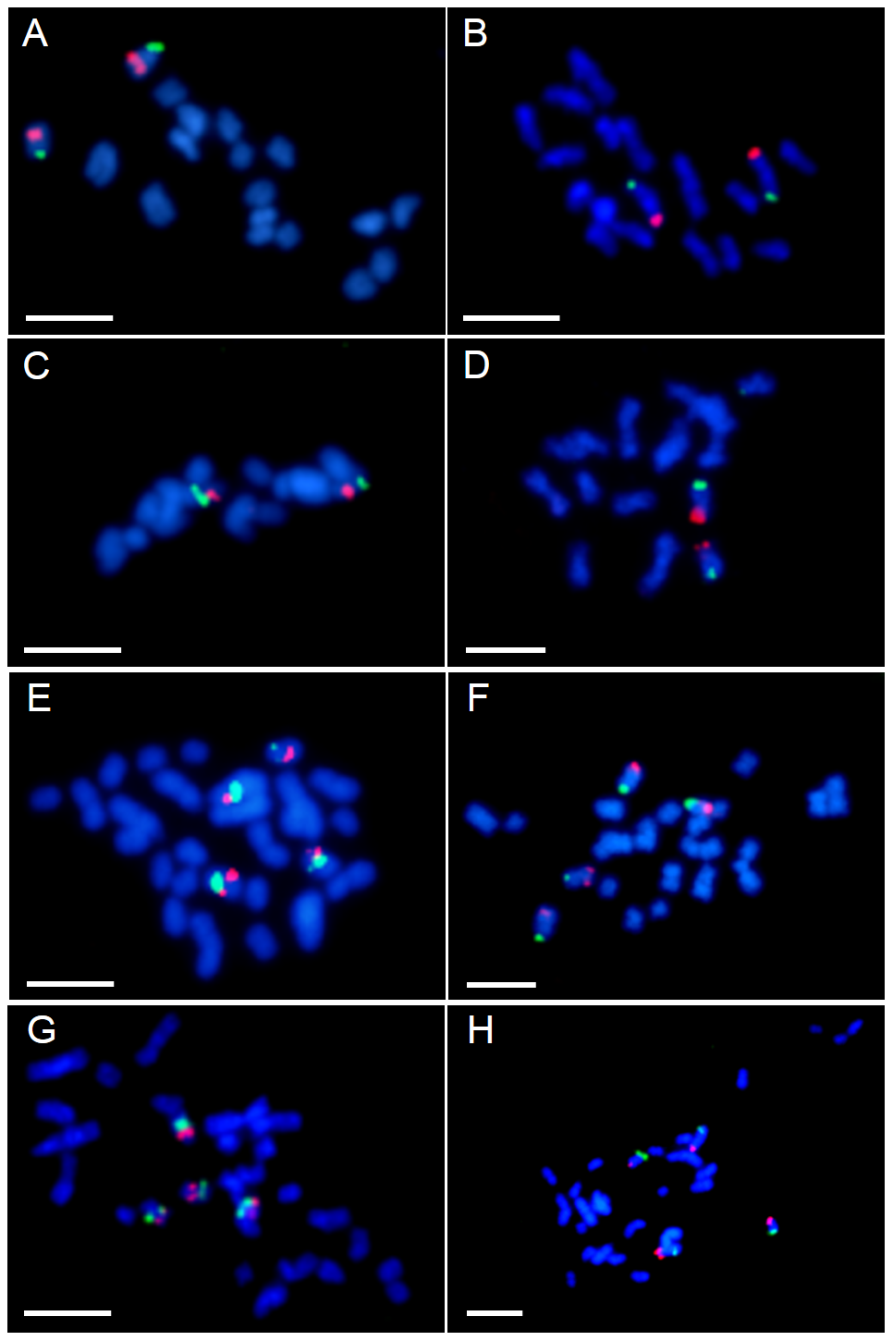
Comparative BAC-FISH mapping of the clones from chromosomes Bd2 and Bd3 to *B. sylvaticum* (A–B), *B. pinnatum* 2n = 18 (C–D), *B. pinnatum* 2n = 28 (E–F) and *B. phoenicoides* (G–H). BACs Bd2/1 – green and Bd2/10 – red (**A**), BACs Bd2/4 – red and Bd2/9 – green (**C, E, G**), BACs Bd3/1 – red and Bd3/6 – green (**B, D, F, H**). Chromosomes were counterstained with DAPI. Bar: 5 µm.

The comparative bioinformatic sequence analyses delivered evidence that chromosome Bd2 arose through the centric fusion of the ancestral equivalents of rice chromosome 1 (Os1) and 5 (Os5) [9]. Clones Bd2/1-4 and Bd2/9-11 are positioned in two separate regions of Bd2, and occupy the distal part of the top and bottom arms (Figure 1) whichcorrespond to different parts of the Os1 equivalent (Figure 4). Additional *in situ* hybridisation performed using a combination of BACs selected from both regions shows that the sets of clones Bd2/1-4 and Bd2/9-11 map to the same chromosome, which is equivalent to Os1 in all of the species analysed (Figure 5A, 5C, 5E and 5G). BAC clones Bd2/5-8 assigned to the region of Bd2 equivalent to Os5 (Figure 4) in all analysed *B. distachyon* relatives map together to a different chromosome from the rest of the clones (Figures 1 and 3).

These results demonstrate that in the karyotypes of *B. distachyon* relatives the homeologues of chromosome Bd2 are represented by two distinct chromosomes that are equivalent to Os1 and Os5 (Figure 4). One or two pairs of each homeologue were found in the diploid and allopolyploid genotypes, respectively. Subtle discrepancies from this general pattern, which were limited to individual clones only, were observed among the species analysed.

### BACs derived from chromosome Bd3

The homeologues of chromosome Bd3 that were identified by comparative BAC-FISH seem to be more stable in the karyotypes of the species studied than the homeologues of chromosome Bd2. Only single hybridisation sites for all of the probes were found in the karyotypes of *B. sylvaticum* and *B. pinnatum* (2n = 18) (Figure 2B–2C). The number of chromosomes discriminated as well as the distribution of the signals of individual BACs were identical in both 2n = 18 diploid species. In the allopolyploid *B. pinnatum* and *B. phoenicoides*, each of the BAC clones had two hybridisation sites, which were located on two separate chromosomes (Figure 2D–2E). As in case of the diploid species, both allopolyploids were very similar regarding the size of the chromosomes and the position of the BAC signals.

**Figure 6:**
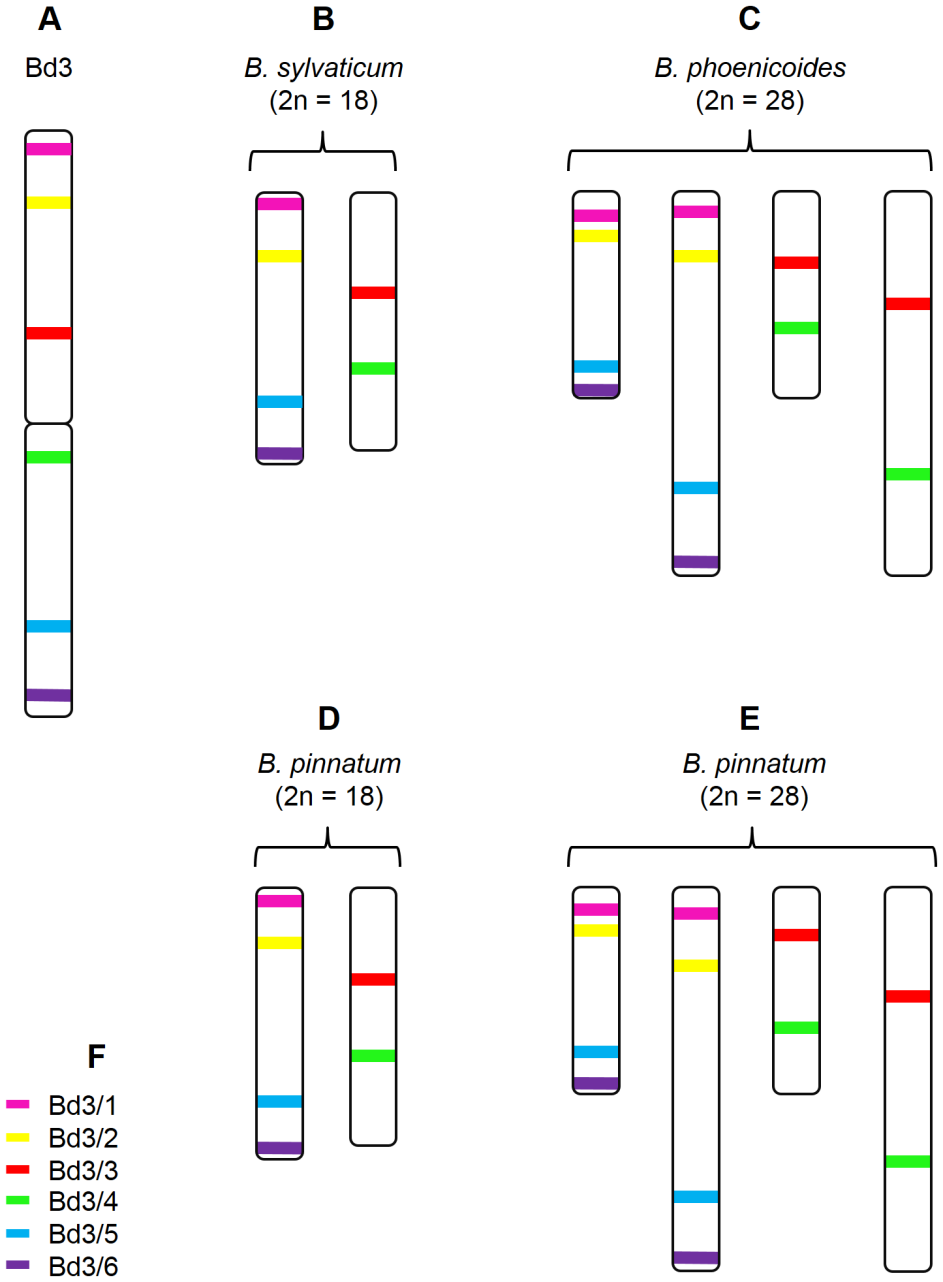
Cytogenetic maps of the chromosomes bearing regions homeologous to the chromosome Bd3 in various *Brachypodium* species. Coloured bars mark the chromosomal positions of specific BAC clones assigned to the chromosome Bd3 in *B. distachyon* (**A**), *B. sylvaticum* (**B**), *B. phoenicoides* (**C**), *B. pinnatum* 2n = 18 (**D**), *B. pinnatum* 2n = 28 (**E**). Colour codes for the clones used in the study (**F**). The position of the clones on the diagram (**A**) reflects their localisation on the physical map [32].

Clones Bd3/1 and Bd3/2 mapped to the same chromosome in all of the species (Figure 2). The BAC signals were located in the terminal and interstitial positions, respectively. In both allopolyploids, the size of the two chromosome pairs that carried the hybridisation signals differed significantly with one pair being nearly twice as long as the other (Figure 2D–2E). Clones Bd3/3 and Bd3/4, which flanked the centromere in *B. distachyon* (Figures 2A and 6A), in the other species landed together on chromosomes that were different from the chromosomes bearing signals for Bd3/1 and Bd3/2. The hybridisation sites of these clones were located on opposite chromosome arms in proximal positions. Also, in this case chromosome pairs discriminated by this combination of BACs in the allopolyploids displayed a striking size difference. A set of complementary experiments revealed that clones Bd3/5 and Bd3/6 map together to the same chromosome as Bd3/1 and Bd3/2 but in opposite arms (Figures 2, 5B, 5D, 5F and 5G).

**Figure 7:**
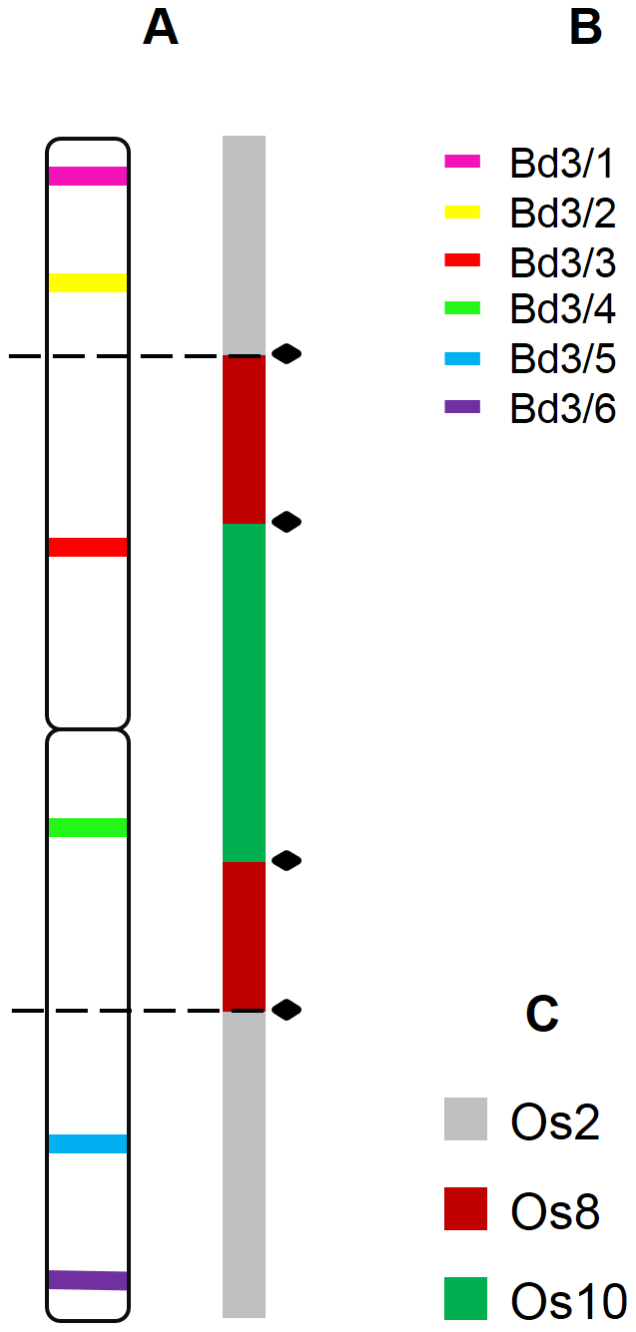
Positions of the clones used in reference to the regions of the chromosome Bd2 homeologous with rice chromosomes. Coloured bars mark the chromosomal positions of specific BAC clones assigned to the chromosome Bd3. A cytogenetic map of *B. distachyon* chromosome Bd3 linked with the Bd3 assembly schematics showing the regions that correspond to different rice chromosomes (**A**) (adapted from [9]), Colour codes for the clones used in the study (**B**), Colour codes for the rice chromosomes homologous to the chromosome Bd3 (**C**). Black diamonds identify the positions of the fusion points in the Bd3. Dashed lines mark the chromosomal breakpoints found in *B. sylvaticum*, *B. pinnatum* 2n = 18 and 2n = 28 and in *B. phoenicoides*. The position of the clones on the diagram (**A**) reflects their localisation on the physical map [32].

The structure of chromosome Bd3 of *B. distachyon* is the result of two dysploidy events that involved three ancestral chromosomes equivalent to rice chromosomes Os2, Os8 and Os10 [9], [19]. According to bioinformatic data, the equivalent of Os10 occupies the most internal position in the chromosome and is flanked by the equivalent of Os8, which is itself inserted into the Os2 equivalent (Figure 7). In the diploid relatives of *B. distachyon*, chromosome Bd3 has two homeologous counterparts (Figure 2B–2C and 6). This indicates that only one dysploidy step occurred during the evolution of the *B. pinnatum* (2n = 18) and *B. sylvaticum* karyotypes. The proximal positions of clones Bd3/3 and Bd3/4 in one of the homeologues suggest that the putative centric fusion comprises the equivalents of Os10 and Os8 (Figures 6 and 7). The other homeologue that bears the sequences of clones Bd3/1-2 and Bd3/5-6 would thus be the equivalent of Os2. However, to fully confirm this hypothesis, additional experiments using clones assigned to the region of Bd3 that are homeologous to Os2 would be necessary. Interestingly, the inferred dysploidy step that was observed in the genomes of diploid *B. distachyon* relatives is also shared by both parental genomes of the allopolyploid *B. pinnatum* as well as by the putative ancestors of *B. phoenicoides*.

## Discussion

### Karyotype evolution in *Brachypodium* species

The evolution of eudicot and monocot lineages is driven by two counteracting processes: whole genome duplication (WGD) and subsequent diploidisation [5], [7], [9], [18]. Chromosome reshuffling constitutes an important part of the diploidisation as it contributes to maintain the proper functioning of the nuclear genome in a newly arisen polyploid. In the case of monocots, it is inferred that all of the present grass genomes evolved from an intermediate ancestor with 12 chromosomes which itself arose from a 5- (or 7-) chromosome ancestor via a WGD and two reciprocal translocation events [7], [21]. Nested chromosome fusions played an important role in the further divergence of grass genomes from an intermediate ancestor. Although this particular rearrangement is common in grasses, it rarely occurs in eudicots, in which end-to-end fusions are mostly responsible for a reduction in the chromosome number [3], [22], [33]. Interestingly, the rice karyotype did not undergo any nested chromosome fusions. It has the highest degree of resemblance to the intermediate ancestral karyotype and thus might serve as a reference for describing the macrosynteny between present species and their relationship to the paleogenomes of the grass ancestors. Triticeae genomes with a basic chromosome number of 7 were formed through five centric chromosome fusions, while seven nested fusions were required to reach the present 5-chromosome karyotype structure in *B. distachyon* [9], [34]. The fusions in the genomes of Triticeae and *B. distachyon* involved different combinations of ancestral chromosomes thus suggesting that they were independent of each other [35]. The diploid *Brachypodium* species that were analysed in the present study have n = 9 chromosomes in their karyotypes, which suggests that three chromosome fusions must have taken place during the divergence from their 12-chromosome ancestor. By barcoding the chromosomes of *B. sylvaticum* and *B. pinnatum* with BAC clones derived from *B. distachyon* genomic libraries, we attempted to determine whether the pattern of descending dysploidy events was shared between these species and *B. distachyon* or whether it occurred independently. It seems that, with regard to the structure of Bd2 and Bd3 homeologues, the karyotypes of *B. sylvaticum* and *B. pinnatum* show a high degree of similarity. Both species lack the fusion between the equivalents of rice chromosomes Os1 and Os5 that was observed in *B. distachyon* but share the nested insertion of the Os10 equivalent into Os8 equivalent. The latter dysploidy step also occurred in *B. distachyon* as a part of an Os10-Os8-Os2 fusion that is specific to this species. A similar observation of *B. distachyon* chromosomes Bd1 and Bd4 was made by Wolny et al. [27]. Each of these chromosomes arose as a result of two fusions that involved ancestral equivalents of Os3, Os7 and Os6 in the case of Bd1, and Os12, Os9 and Os11 in the case of Bd4. All of the diploid relatives of *B. distachyon* studied in Wolny et al. [27] were characterised by the presence of Os3-Os7 and Os9-Os11 fusions, thus indicating that the presence of three-chromosome configurations that involves the equivalents of Os6 and Os12, respectively, is a unique feature of the *B. distachyon* genome.

Although the first phylogenetic analyses gave *B. distachyon* a basal position in the genus [36], it has been stated recently that *B. distachyon* shares a relatively recent common ancestor with Eurasian perennial species (including *B. sylvaticum* and diploid *B. pinnatum*), and diverged 5 Mya around the same time as the perennials. It is likely that the Os8-Os10, Os3-Os7 and Os9-Os11 fusions occurred in the karyotype of this recent ancestor and that descending dysploidy progressed further in *B. distachyon* through the subsequent insertion of the first fusion products into other ancestral chromosomes as a part of the divergence of its genome.

The correspondence between the positions of BAC clones in the Bd2 and Bd3 homeologues and synteny breakpoints identified in the karyotype of *B. distachyon* gives support for a pattern of nested chromosome insertions that were modelled previously using sequencing data [9]. The combined cytogenetic data from this study and [27] indicate that the dysploidy events that govern the chromosome number in *B. sylvaticum* and diploid *B. pinnatum* are the same. We propose that nine chromosomes of their genomes can be represented by rice equivalents of ancestral chromosomes as follows: Os3-Os7, Os6 (homeologues of chromosome Bd1), Os1, Os5 (homeologues of Bd2), Os8-Os10, Os2 (homeologues of Bd3), Os9-Os11, Os12 (homeologues of Bd4) and Os4 (homeologue of Bd5).

Although both species share the dysploidy pattern, their genomes are differentiated by other chromosomal rearrangements such as duplications, translocations and inversions. For example, the presence of a chromosome pair carrying an additional site for Bd2/1 in the diploid *B. pinnatum* might have resulted either from a duplication or a translocation event. However, the smaller size of the signals and their lower intensity than in the chromosomes with hybridisation sites for both probes support the latter hypothesis. Another interesting example is the specific chromosomal localisation of the clone Bd2/11 in *B. sylvaticum*, which can possibly be explained by a reciprocal translocation between the chromosomes carrying sites for Bd2/10 and Bd2/11.

It is intriguing, though, why the changes detected in our study affected only the homeologues of chromosome Bd2. It is possible that the Bd3 homeologue regions that contain the BACs used in the study were not involved in additional reshuffling, and that some structural rearrangements could have been found with a different choice of clones for mapping. However, it cannot be ruled out that for some reason Bd2 homeologues are structurally less stable than Bd3. The occurrence of chromosome regions that are more prone to structural rearrangements was postulated for animals and plants [37]. The position of such hotspots has been ascribed to the presence of transposable elements, segmental duplications, nonallelic homologous recombination hotspots or gene-rich regions containing adaptation traits [37]–[41]. It is possible that the homeologues of Bd2 comprise loci which became preferentially involved in the reshuffling because they could provide an adaptive advantage for the evolving species. Alternatively, one or more of the other factors mentioned above played a role in the chromosomal rearrangements that were observed in the genomes of *Brachypodium* diploids.

### Karyotype structure of *Brachypodium* allopolyploids

A cytogenetic analysis of allopolyploid *B. pinnatum* and *B. phoenicoides* using genomic *in situ* hybridisation (GISH) with total nuclear DNA of different *Brachypodium* diploids indicates that *B. distachyon* is a likely candidate for one of the ancestral species for both polyploids [31]. Moreover, the chromosome number of *B. distachyon* fits the hypothesis that allopolyploids with 2n = 28 chromosomes arose through hybridisation between 2n = 10 and 2n = 18 species. Surprisingly, the subsequent molecular phylogenetic data excluded *B. distachyon* as a putative parent of *B. pinnatum* (2n = 28) and *B. phoenicoides* [27]. The phylogeny based on single-copy nuclear gene sequence analysis points to *B. pinnatum* (2n = 18) and *B. rupestre* (2n = 18) as components of these polyploids. Our data support the molecular phylogenetics findings as no chromosomes structured exactly like Bd2 and Bd3 were found in the allopolyploid karyotypes that were studied using comparative BAC-FISH. The Bd2 homeologues that belong to the two genomes constituting the polyploid complement differ in size, but their structure is nearly identical and resembles the structure of their counterparts in the diploids with 2n = 18. An analogous situation was observed regarding the Bd3 homeologues. The presence of additional signals of the Bd2/1 BAC clone in both the diploid and allopolyploid *B. pinnatum* as well as in *B. phoenicoides* (Figure 1C–E) supports the assumption that *B. pinnatum* (2n = 18) is one of the ancestors, assuming that the observed duplication of the BAC sequence occurred before the hybridisation events. Alternatively, it could be attributed to chromosomal rearrangements that took place in the hybrid genome after the allopolyploidisation event.

If allopolyploid *B. pinnatum* and *B. phoenicoides* resulted from the hybridisation between two species with 2n = 18 chromosomes, their chromosome numbers must have been reduced by dysploidy. If this assumption is true then in order to achieve a reduction of chromosome number from 2n = 36 to 2n = 28, eight dysploidy events were required in addition to the fusions that were already present in the parental genomes. It is yet to be determined whether these fusions are distributed evenly between the component genomes and whether they involve the same ancestral chromosomes thus resulting in the same pattern of dysploidy in both parental karyotypes. In our study, the Bd2 homeologues that carried signals of BACs Bd2/5-8 in *B. pinnatum* (2n = 28) differed significantly in size and position (Figures 1D and 3D). It is possible that the larger of the homeologues underwent additional fusion thereby generating the interstitial position of Bd2/5 and Bd2/6, whilst the smaller homeologue was not involved in the ongoing dysploidy. Another example of BAC-FISH pattern polymorphism in the polyploids is the simultaneous presence of two configurations of Bd1 homeologues: one comprising an Os3-Os7 fusion product and an Os6 equivalent, and one comprising an Os6-Os7 fusion and an Os3 equivalent [27]. These data clearly indicate that dysploidy events in the component genomes are not necessarily uniform and can differ in terms of the quality and quantity of the fusions both preceding and following the hybridisation events.

## Conclusions

A progressive reduction in chromosome number is observed within the *Brachypodium* genus, which comprises species with x = 10, 9, 8, 7 or 5. Using comparative BAC-FISH barcoding of chromosomes Bd2 and Bd3, we identified descending dysploidy events that are common for the species with x = 9 and *B. distachyon*, as well as chromosome fusions specific only for the *B. distachyon* karyotype. Our results appear to support the hypothesis that dysploidy events that are shared by different lineages of the genus appeared earlier in their common ancestor. However, independent chromosome fusions cannot be excluded. It still remains to be determined whether the pattern of fused chromosomes is shared between other *Brachypodium* species. To answer this question, future studies should include other species with 2n = 18 such as *B. rupestre*, *B. arbuscula* or *B. flexum* [30] as well as species with different chromosome numbers.

The well-developed cytogenetic platform for *Brachypodium* species permits two complementary approaches to comparative mapping – chromosome barcoding and chromosome painting. The cytogenetic data presented here are consistent with the localisation of synteny breakpoints between *B. distachyon* and rice that was inferred from the bioinformatic analysis of genomic sequences [9]. Using painting probes that are based on synteny breakpoints should be particularly valuable for the reconstruction of the evolutionary history of the extant karyotypes in the genus *Brachypodium*.

## Materials and Methods

### Plant material

Four *Brachypodium* species were selected for comparative BAC-FISH analysis. *Brachypodium* accessions were sourced from the collections held by the Aberystwyth University, UK and USDA-NPGS. The names of the species that were analysed along with information about their origin and number of chromosomes are shown in Table 1.

### Chromosome preparations

Mitotic chromosome preparations were made as described in detail in Jenkins and Hasterok [42] with minor modifications. The seeds were germinated for 4–5 days in the dark in Petri dishes on filter paper moistened with distilled water. Seedlings with roots 1.5–2.0 cm long were immersed in ice-cold water and incubated for 24 hours at 4°C in order to accumulate metaphases. After this treatment, the seedlings were fixed in 3∶1 methanol/glacial acetic acid at room temperature for several hours and then stored at −20°C until required.

Excised root tips were washed three times in 0.01 M citric acid-sodium citrate buffer (pH 4.8), 5 minutes each time and digested in a mixture of enzymes comprising 4% pectinase (Sigma, St Louis, MO, USA P5146), 1% cellulose (Calbiochem, San Diego, CA, USA, 21947) and 1% cellulase ‘Onozuka R-10’ (Serva, Heidelberg, Germany 16419) for 1.5 hour at 37°C. Multi-substrate preparations were made according to the procedure described by Hasterok et al. [43]. Meristems of three different species of *Brachypodium* were dissected from the root tips and transferred separately in a small volume of 45% acetic acid followed by the arrangement of the digested material on a slide to form a triangle. Slides were covered with coverslips, gently squashed and frozen on dry ice. After freezing and coverslip removal, the slides were air-dried and stored at 4°C until used.

### BAC clone selection

The BAC clones used in the study originated from two *B. distachyon* genomic libraries [9], [32]. Two sets of BACs assigned to *B. distachyon* chromosomes Bd2 and Bd3 were selected from the assemblies of FPCs (FingerPrinted Contigs) that had previously been aligned to the *B. distachyon* karyotype [26], [32]. The clones in each set were selected to be distributed along the entire length of a given chromosome and to contain very low amounts of repetitive DNA. Preliminary FISH experiments showed that one of the clones that had localised proximally in the bottom arm of chromosome Bd2 yielded non-specific hybridisation signals in the centromeres of all of the chromosomes. This clone was excluded from further studies. Finally, 11 and 6 clones assigned to chromosome Bd2 and Bd3, respectively, were chosen for comparative chromosome mapping. Each clone was mapped to the preparations derived from approximately ten individual plants. The list of BACs used and their characteristics are shown in Table 2.

### Probe labelling and FISH

DNA from each BAC clone was isolated by standard alkaline extraction as described by Farrar and Donnison [44] and subsequently labelled by nick–translation using tetramethylrhodamine-5-dUTP (Roche, cat. no. 11534378910) or digoxigenin-11-dUTP (Roche, cat. no. 11093088910) according to the protocols by Jenkins and Hasterok [42]. Pairs of differentially labelled BACs were mapped to multi-substrate chromosome preparations. Each of the clones was mapped in combination with the preceding and the following clone.

The FISH procedure followed the protocol published by Jenkins and Hasterok [42] with some modifications. Slides were pre-treated with RNase (100 µg/ml) in 2× saline sodium citrate (SSC) at 37°C for 1 hour, washed several times in 2× SSC, dehydrated in ethanol and air dried. For heterologous BAC-FISH, a low-stringency hybridisation mixture containing 30% deionized formamide, 40% dextran sulphate, 2× SSC, 1% SDS, and 2.5–3.0 ng/ml of each DNA probe was prepared. The hybridisation mixture with probes was predenatured at 75°C for 10 minutes, applied to the slides and denatured together with chromosome preparations at 75°C for 4.30 minutes. Hybridisation was performed in a humid chamber at 37°C for 16–20 hours. Post-hybridisation washes were performed in 20% deionised formamide in 2× SSC at 37°C, which is equivalent to 59% stringency.

Probes labelled with digoxigenin-11-dUTP were detected using a fluorescein isothiocyanate-conjugated anti-digoxigenin antibody (Roche, cat. no. 11207741910). Probes labelled with tetramethylrhodamine-5-dUTP were directly visualised. The preparations were mounted and counterstained in a VectaShield antifade solution (Vector Laboratories Burlingame, CA, USA) containing 2.5 µg/ml DAPI (Serva).

### Image acquisition and processing

Photomicrographs were taken using a monochromatic CCD camera attached to a Provis AX wide-field epifluorescence microscope (Olympus) using the respective narrow band filter sets. All images were processed uniformly and superimposed using Photoshop CS3 (Adobe).
